# NAT10-mediated N4-acetylcytosine modification promotes the progression of retinoblastoma by improving the HK1 mRNA stability to enhance glycolysis

**DOI:** 10.1016/j.clinsp.2025.100678

**Published:** 2025-05-08

**Authors:** Shan Xu, Xuming Lin, Fengling Jia

**Affiliations:** Department of Ophthalmology, Yantai Yuhuangding Hospital, Yantai, Shandong, China

**Keywords:** Retinoblastoma, ac^4^C modification, NAT10, HK1, Glycolysis

## Abstract

**Objective:**

Retinoblastoma (RB) is a type of intraocular tumor in childhood with a high lethality rate. N4-acetylcytosine (ac^4^C) modification is known to regulate multiple cancers, which is mediated by the only known ac^4^C writer N-Acetyltransferase 10 (NAT10). In this study, the authors aimed to reveal the mechanism of RB progression regulated by ac^4^C modification.

**Method:**

Phenotypically, dot blot assay and quantitative real-time PCR were used to detect the ac^4^C levels and NAT10 expression in clinical samples and RB cell lines. Then, NAT10 was knocked down to assess its effect on glycolysis. Mechanically, RNA immunoprecipitation assay, immunofluorescence assay, and dual luciferase report were used to explore the mRNA modified by NAT10-mediated ac^4^C modification. Mice xenograft model was used to determine the effect of NAT10 on tumor growth *in vivo*.

**Results:**

The present results demonstrated that the levels of ac^4^C and NAT10 were increased in cancer tissues and RB cell lines. Furthermore, NAT10 knockdown inhibited the glycolysis in RB cell lines. Moreover, the authors revealed that NAT10 knockdown decreased the ac^4^C modification and mRNA stability of HK1, while the inhibition of glycolysis by NAT10 knockdown was reversed by HK1 overexpression. Finally, NAT10 knockdown relieved the growth of tumors in mice models.

**Conclusion:**

The authors illustrated that NAT10 plays an important role in the progression of RB by regulating the ac^4^C modification on HK1 mRNA and affects its stability, which may provide a novel theoretical basis for the treatment of RB.

## Highlights

• NAT10 mediated ac^4^C modification by interacting with HK1.
• The levels of ac^4^C and NAT10 were increased in cancer tissues and RB cell lines.
• NAT10 knockdown inhibited the glycolysis in RB cell lines.

## Introduction

Retinoblastoma (RB) is the most common malignant intraocular tumor in childhood.^1^ with a high mortality rate. The pathogenesis of RB is caused by the mutations of the RB1 gene, which is a tumor suppressor gene that leads to the loss of function of RB proteins and affects the cell cycle, eventually causing abnormal cell proliferation.^2^ In addition, the lack of RB proteins is reported to alter metabolic pathways to provide the energy for tumor growth.^3^^,^^4^ It has been demonstrated that glycolysis is elevated in RB.^5^ Moreover, Babu et al.^6^ confirmed that retinoblastoma tumors and *in vitro* models lack Hexokinase-1 (HK1) and exhibited elevated fatty acid oxidation, which might be taken as an indicator of the progression of RB.

In recent years, numerous studies have reported that epigenetics is involved in the development of cancer, including methylation, acetylation, and phosphorylation.^7^, ^8^, ^9^ Furthermore, RB tumor procession mediated by RNA modification has been reported in some studies. For example, NSUN2-mediated 5-methylcytosine (m^5^C) RNA methylation promotes the stability and expression of PFAS mRNA to regulate the RB progression.^10^ At present, the RNA modification regulation of RB progression mainly focuses on the study of N6-methyladenosine (m^6^A), and the study of other modifications remains to be explored.^11^^,^^12^

N^4^-acetylcytosine (ac^4^C) is a newly discovered RNA modification, due to the limited research on ac^4^C, it is the only acetylation event described in eukaryotic RNA in current studies.^13^^,^^14^ N-Acetyltransferase 10 (NAT10), an essential ATP-dependent enzyme, is also the first enzyme to be found to catalyze ac^4^C production in eukaryotic RNA, which has acetyltransferase activity and RNA-binding activity.^15^^,^^16^ NAT10 is involved in the progression of many cancers by regulating ac^4^C modification, and the mechanism has been widely reported recently. Zhang et al.^17^ first demonstrated that NAT10 inhibits ferroptosis through ac^4^C and stabilization of Ferroptosis Suppressor Protein-1 (FSP1) mRNA to promote colon cancer progression. Wang et al.^18^ revealed that the inhibition of NAT10 led to the restrain of ac^4^C and repressed the mRNA's stability and protein expression thus preventing the procession of bladder cancer. However, the function of NAT10 in mediating RB progression by regulating ac^4^C modification remains unclear yet.

To explore the mechanism of ac^4^C modification regulated by NAT10 on RB progression, the authors detected the expression of ac^4^C and NAT10 in RB clinical samples and RB cell lines. The underlying mechanism was assessed using *in vitro* experiments, and the role *in vivo* was analyzed using a cell-derived xenograft mouse model. These results may provide a new theoretical basis for the study of RB progression and a potential therapy of RB.

## Methods

### Ethical statement

This study was performed in line with the principles of the Declaration of Helsinki. Approval was granted by the Ethics Committee of Yantai Yuhuangding Hospital (Approval no. 2025-075). Written informed consent was obtained from the legal guardians. All methods were carried out in accordance with relevant guidelines and regulations.

### Sample collection

RB tissue specimens (30 cases) and corresponding adjacent ones (30 cases) from the hospital were collected from patients with RB and stored in liquid nitrogen for use. All tissue samples were verified by pathological examination. The following patients were excluded: 1) Those who received chemotherapy or radiotherapy and 2) Those who had other cancers. Written informed consent was obtained from the legal guardians, and this study was approved by the hospital (Approval no. 2025-075).

### Cell culture and treatment

The adult retinal pigment epithelium cell line ARPE-19, the RB cell lines Y-79 and WERI-Rb1, and 293T cells were provided from ATCC (Manassas, VA, USA). ARPE-19, Y-79 and WERI-Rb1 cells were cultured in RPMI-1640 medium (Gibco, Grand Island, NY, USA) and 293T cells were cultured in DMEM (Gibco) respectively supplemented with 10 % fetal bovine serum and 1 % penicillin/streptomycin at 37 °C and 5 % CO_2_.

### Dot blot assay

Total RNA was extracted using Trizol reagent (Invitrogen, Carlsbad, CA, USA) and diluted in 10 mM Tris-EDTA buffer. Then, RNA samples were loaded onto Hybond-*N*+ membranes (Solarbio, Beijing, China), and the membrane was crosslinked at 254 nm UV for the 60 s after a short drying process, blocked with 5 % milk for 1.5 h. Afterward, the membrane was incubated with anti-ac^4^C (1:250, ab252215, Abcam, Cambridge, UK) overnight at 4 °C and then incubated with a secondary antibody (1:10,000, ab6721, Abcam) for 1.5 h. Finally, blots were visualized using an enhanced chemiluminescence kit (Thermo Scientific, Waltham, MA, USA).

### Quantitative real-time PCR (qRCR)

Total RNA was extracted using Trizol reagent (Invitrogen) and the cDNA was synthesized using a PrimeScript RT reagent kit (Takara, Tokyo, Japan). The cDNA was analyzed by qPCR on the ABI-7500 PCR instrument (Applied Biosystems, Waltham, MA, USA) using the SYBR qPCR master mix kit (Invitrogen). The expression of NAT10 and HK1 were calculated by the 2^-∆∆Ct^ method with GAPDH as the endogenous reference. The primers for qPCR were as follows: NAT10, 5′-ATAGCAGCCACAAACATTCGC-3′ (sense) and 5′-ACACACATGCCGAAGGTATTG-3′ (antisense); HK1, 5′-GCTCTCCGATGAAACTCTCATAG-3′ (sense) and 5′-GGACCTTACGAATGTTGGCAA (antisense).

### Cell transfection

Short hairpin RNA targeting NAT10 (shNAT10), shRNA negative control (shNC), empty vector (pcDNA3.1), and HK1 overexpressing plasmid (pcDNA3.1-HK1) were acquired from GenePharma (Shanghai, China). Cell transfection was implemented by utilization of Lipofectamine 3000 (Invitrogen) in line with the directions recommended by the manufacturer.

### Cell viability

The effect of NAT10 knockdown on Y-79 and WERI-Rb1 cell viability was counted by Cell Counting Kit-8 (CCK-8) (Yeasen, Shanghai, China). Cells were incubated in 96-well plates at a density of 1 × 10^3^ cells/well with 10 μL CCK8 solution for 2 h. After incubation, a microplate reader was used to measure the absorbance at a wavelength of 450 nm.

### Relative glucose consumption

The glucose consumption was measured using a glucose assay kit (Beyotime, Shanghai, China) after Y-79 and WERI-Rb1 cells were transfected for 24 h. Cells were lysed by the cell and tissue lysis buffer (Beyotime) and centrifuged for 5 min at 12,000 g to get supernatant. Then, supernatant (5 μL) and glucose assay reagent (185 μL) were added to 96-well plates, heated on PCR apparatus at 95 °C for 8 min, and cooled down to 4 °C. The absorbance was measured at 630 nm by a microplate reader.

### Relative lactate production

The lactate production was measured by a lactic acid content assay kit (Sangon, Shanghai, China) after Y-79 and WERI-Rb1 cells were transfected for 24 h. Cells were broken by ice bath ultrasound for 3 min and centrifuged for 10 min at 12,000 g to get supernatant. Supernatant and reagents were sequentially added to 96-well plates according to guidelines and reacted in a water bath at 37 °C for 20 min. Then, other reagents were added according to the guidelines and reaction for 20 min at 37 °C protected from light. After the reaction, the deposits were obtained by centrifuging for 10 min at 10,000 g. Finally, the deposits were dissolved by 1000 μL alcohol, and the absorbance was measured at 570 nm by a microplate reader.

### Real-time cell metabolism assay

To determine the effects on glycolysis of NAT10 knockdown and HK1 overexpression in Y-79 and WERI-Rb1 cells, the Extracellular Acidification Rate (ECAR) and the Oxygen Consumption Rate (OCR) were measured using an XFp extracellular flux analyzer (Seahorse Bioscience, North Billerica, MA, USA). The cells were resuspended in sterile XF base media supplemented with 10 mM d-glucose (pH 7.4) and settled at 37 °C for 30 min. The OCR was measured after injections of 0.5 µM oligomycin, 1 µM FCCP, 1 µM antimycin A, and 1 µM rotenone. The ECAR was quantified after 10 mM glucose, 1 µM oligomycin, and 50 mM 2-deoxyglucose were injected.

### RNA immunoprecipitation (RIP) assay

RIP assay was performed using an imprint RNA immunoprecipitation kit (Sigma-Aldrich, St. Louis, MO, USA). To evaluate the interaction between NAT10 and HK1, magnetic protein A/G beads pre-coated with anti-HK1 (1:1000, ab150423, Abcam) or anti-IgG were incubated with cell lysates at 4 °C overnight. After purification, RNA was isolated by Trizol and detected by qPCR.

To measure the ac^4^C levels of glycolysis-related mRNAs, total RNA was extracted from 293T cells by Trizol. Magnetic protein A/G beads were coated with anti-ac^4^C (1:250, ab252215, Abcam) or anti-IgG and incubated with total RNA. The mRNA levels of G6PC2, HKDC1, ALDOC, ENO2, and HK1 were measured by qPCR after purification.

### Immunofluorescence assay

The cells were seeded onto coverslips in 6-well plates and incubated for 12 h. Afterward, the cells were fixed with immunostaining fix solution and permeabilized with 0.2 % Triton X-100 in phosphate-buffered saline (PBS, Gibco). Next, the cells were washed with PBS and 3 % bovine serum albumin (blocking reagent) for 1 h. Then, the cells were incubated with anti-HK1 (68,419–1-Ig, 1:2000, Proteintech Group, Rosemont, IL, USA) and anti-NAT10 (ab194297, 1:2000, Abcam) for 1 h. Finally, fluorescent secondary antibodies (ab150115 for HK1 and ab150077 for NAT10, 1:1000, Abcam) were used to incubate with cells for 1 h in the dark. The nuclei were counterstained with DAPI (1 μg/mL, Beyotime), and cells were observed under a confocal microscope.

### Dual luciferase reports

The two luciferase reporter plasmids, wild-type (wt)-HK1 and mutant (mut)-HK1 were constructed. For luciferase assays, 293T cells were seeded in 24-well plates at a concentration of 1 × 10^5^ cells/well. The cells were co-transfected with the wt-HK1 or mut-HK1 plasmid and shNC or shNAT10 with Lipofectamine 3000 for 24 h. Then, the luciferase activity in the harvested cells was measured using the dual-luciferase reporter assay system (Promega, San Luis Obispo, CA, USA).

### RNA stability

293T cells were treated with 5 μg/mL actinomycin D (Merck) for 0 h, 6 h, 12 h, 18 h and 24 h in 6‐well plates. The HK1 mRNA level was estimated by qPCR analysis as mentioned above.

### Cell-derived xenograft mouse models

Six to eight-week-old BALB/c nude mice were randomly divided into two groups, shNC (*n* = 6) and shNAT10 (*n* = 6). Y-79 cells transfected with shNAT10 or shNC (5 × 10^6^ cells) were injected subcutaneously into the mice. The first week was considered when the tumor volume reached about 100 mm^3^. Tumor volumes were measured from 1 to 5 weeks (once a week) using a vernier caliper and calculated as V = (length)  ×  (width)  ×  (height)  ×  0.52.^19^ Five weeks later, mice were euthanized using sodium pentobarbital (i.p.) at the concentration of 160 mg/kg, and tumors were extracted.

### Immunohistochemistry staining

Tissues from mouse models were fixed in formalin, dehydrated in alcohol, and embedded in paraffin (all bought from Aladdin, Shanghai, China). Antigen retrieval was achieved by microwave oven procedure in 10 mmoL/L Tris-EDTA buffer (pH 9.0, Beyotime) after dewaxing and rehydration of the paraffin-embedded sections. Sections were incubated for overnight with primary antibodies anti-HK1 (ab150423, 1:50, Abcam) and anti-Ki67 (ab16667, 1:200, Abcam) at 4 °C. Afterwards, sections were incubated at room temperature with a secondary antibody (ab205718, 1:2000, Abcam) for 30 min. 3,3′-diaminobenzidine (Merck, Darmstadt, Germany) was used as a chromogen. The slides were counterstained with hematoxylin (Merck) and observed using a microscope.

### Statistical analysis

Statistical analysis of data was processed using the SPSS 22.0 software, and the results are shown as mean ± SD. Comparisons were analyzed using one-way analysis of variance (ANOVA) or *t*-test; *p* < 0.05 was considered statistically significant.

## Results

### The ac^4^C modification and NAT10 expression are elevated in RB

**Fig. 1:**
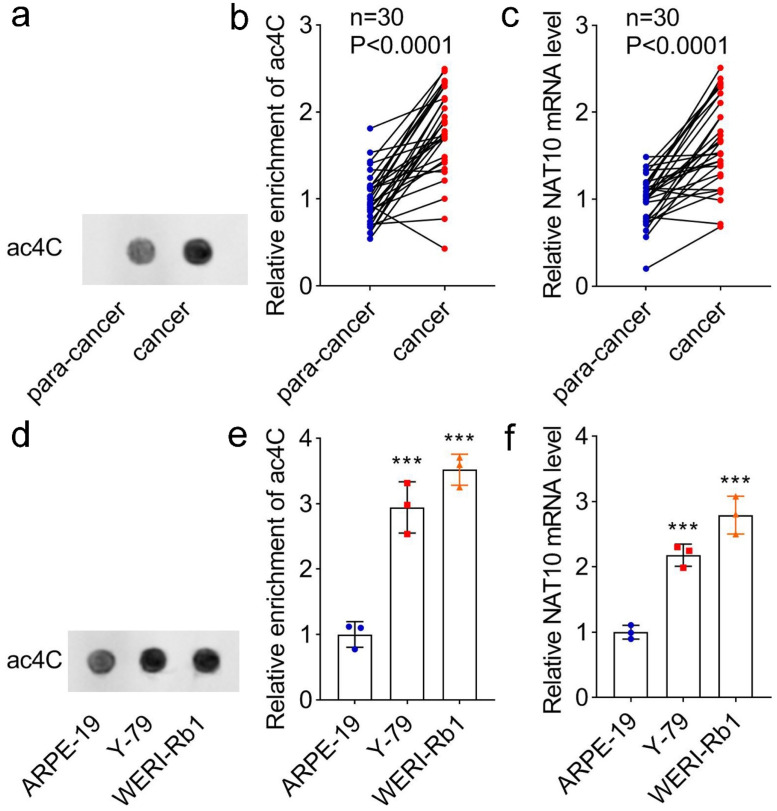
**The levels of ac^4^C and NAT10 in RB clinical samples and RB cell lines.** (a‒b) The ac^4^C levels in cancer tissues and para-cancer tissues were measured by dot blot assay. (c) The NAT10 mRNA levels in cancer tissues and para-cancer tissues were measured by qPCR. (d‒e) The ac^4^C levels in ARPE-19 (retinal pigment epithelium cell line), Y-79 and WERI-Rb1 (RB cell lines) were measured by dot blot assay. (f) The NAT10 mRNA level in ARPE-19, Y-79 and WERI-Rb1 was measured by qPCR. *** *p* < 0.001 vs. ARPE-19 cells.

To explore the function of ac^4^C modification in the progression of RB, the authors compared the modification levels of ac^4^C between cancer and para-cancer samples. As shown in the dot blot assay, the ac^4^C level in cancer tissues was higher than that in para-cancer tissues (Fig. 1a‒b). Additionally, the mRNA expression of NAT10 was increased in cancer tissues (Fig. 1c). Furthermore, RB cell lines (Y-79 and WERI-Rb1) exhibited high levels of ac^4^C modification compared with normal retinal cell line (ARPE-19) (Fig. 1d‒e), as well as the mRNA level of NAT10 in RB cell lines was higher than that in normal retinal cell line (Fig. 1f). These results suggested that the ac^4^C modification and NAT10 expression were elevated in RB, indicating that the increase of NAT10 expression may promote the ac^4^C modification in RB.

### Knockdown of NAT10 inhibits glycolysis

**Fig. 2:**
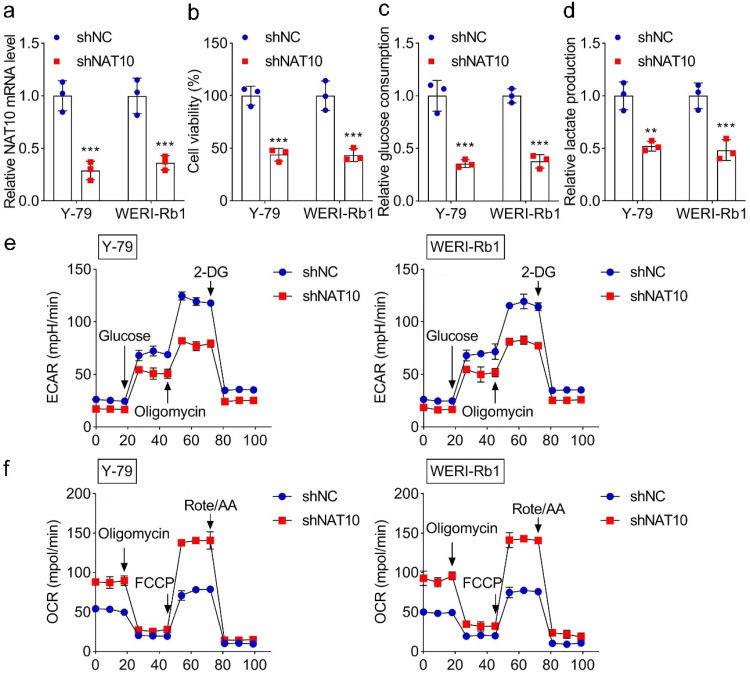
**The effect of NAT10 knockdown on glycolysis.** (a) The expression of NAT10 of Y-79 and WERI-Rb1 transfected with shNAT10. (b) The effect of NAT10 knockdown on cell viability. The effect of NAT10 knockdown on (c) glucose consumption and (d) lactate production. The effect of NAT10 knockdown on (e) ECAR and (f) OCR. ** *p* < 0.01 and *** *p* < 0.001 vs. shNC.

As the authors mentioned above, NAT10 was highly expressed in RB cell lines. To explore its function in RB procession, the authors knocked down the expression of NAT10 in Y-79 and WERI-Rb1 cells using shNAT10 transfection. As shown in Fig. 2a, the NAT10 mRNA level was reduced following the transfection of shNAT10 compared with the shNC group. The knockdown of NAT10 also decreased the cell viability significantly (Fig. 2b). To determine the effect of NAT10 knockdown on glycolysis, the authors first evaluated cell respiration by measuring glucose consumption and lactate production of RB cell lines. The results showed that NAT10 knockdown reduced glucose consumption and decreased lactate production, indicating that NAT10 knockdown inhibited the anaerobic respiration of RB cells (Fig. 2c‒d). In addition, NAT10 knockdown decreased ECAR (Fig. 2e) and elevated OCR (Fig. 2f) in both Y-79 and WERI-Rb1 cells, suggesting that NAT10 knockdown inhibited the glycolysis in RB cells. In summary, NAT10 knockdown inhibited glycolysis in RB cells, which may inhibit RB progression.

### NAT10 mediates ac^4^C modification through interaction with HK1

**Fig. 3:**
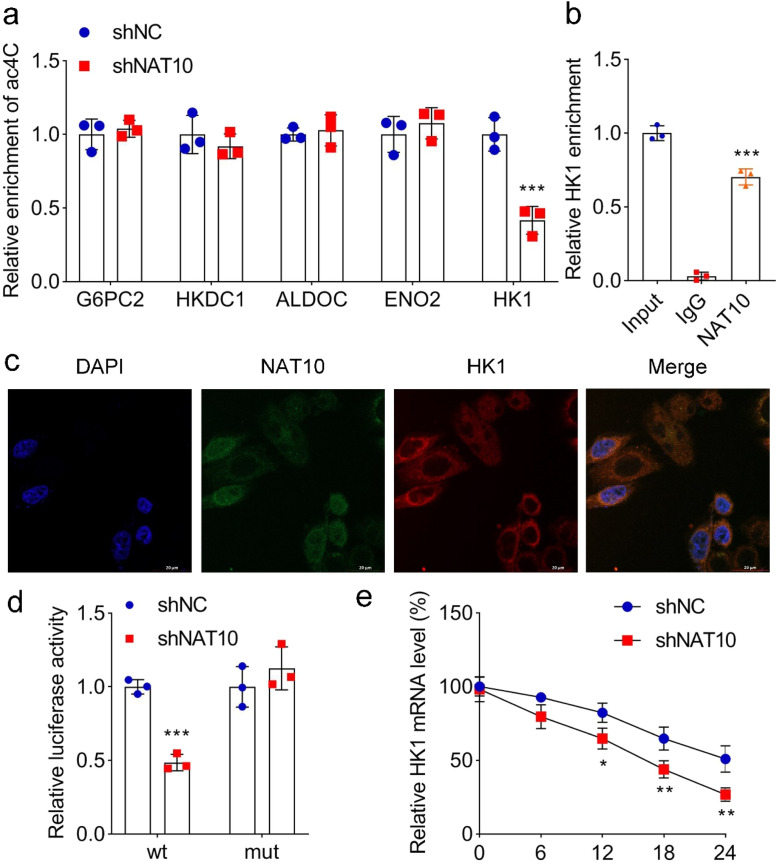
**NAT10 mediated ac^4^C modification by interacting with HK1.** (a) The ac^4^C levels of glycolysis-related genes in 293T cells with NAT10 knockdown. (b) RIP was used to determine the interaction between NAT10 and HK1. (c) The subcellular localization of NAT10 and HK1 was identified by immunofluorescence assay. (d) The luciferase activity of HK1-wt and HK1-mut in 293T cells with or without NAT10 knockdown. (e) HK1 mRNA level in NAT10 knockdown 293T cells with 5 μg/mL actinomycin D treatment for 0 h, 6 h, 12 h, 18 h, and 24 h was measured by qPCR. * *p* < 0.05, ** *p* < 0.01 and *** *p* < 0.001 vs. shNC or IgG.

In search of the target of NAT10 in regulating ac^4^C modification, the authors measured the ac^4^C levels of glycolysis-related genes mediated by NAT10. The results showed that NAT10 knockdown only decreased the ac^4^C level of HK1, while that of other genes were not affected by NAT10 (Fig. 3a). RIP assay proved that NAT10 was combined with HK1 (Fig. 3b). Moreover, the authors identified the subcellular localization of NAT10 and HK1 by immunofluorescence. The results suggested NAT10 was located in the both nucleus and cytoplasm, while HK1 was mainly located in the cytoplasm, with some overlap between them (Fig. 3c). Furthermore, NAT10 knockdown decreased the luciferase activity of wt-HK1, but had no effect on mut-HK1 (Fig. 3d). Overall, the authors demonstrated that there was an interaction between NAT10 and HK1. In addition, after adding actinomycin D to 293T cells, the RNA stability of HK1 affected by NAT10 was measured. The results showed that NAT10 knockdown accelerated the degradation of HK1, suggesting that NAT10 promoted the stability of RNA (Fig. 3e). Taken together, the authors found out that NAT10 mediated ac^4^C modification through interaction with HK1.

### Overexpression of HK1 reverses the inhibition of glycolysis mediated by NAT10 knockdown

**Fig. 4:**
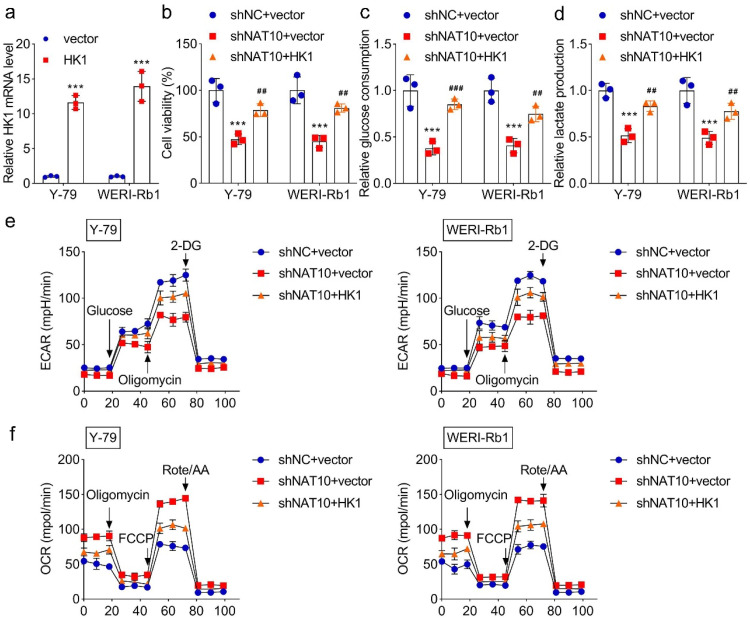
**The effect of HK1 overexpression on glycolysis.** (a) HK1 expression was detected using qPCR following pcDNA3.1 (vector group) and pcDNA3.1-HK1 (HK1 group) transfection. (b) The effect of overexpression on cell viability in RB cell lines. The effect of HK1 overexpression on (c) glucose consumption and (d) lactate production. The effect of HK1 overexpression on (e) ECAR and f. OCR. *** *p* < 0.001 vs. shNC. ## *p* < 0.01 and ### *p* < 0.001 vs. the shNAT10+vector group.

Subsequently, whether NAT10 affected HK1 to regulate glycolysis in RB cell lines was assessed through rescue experiments. To overexpress HK1, pcDNA3.1-HK1 was transfected into Y-79 and WERI-Rb1 cells, and the expression of HK1 in the cells was increased (Fig. 4a). Then, the authors found that overexpression of HK1 significantly increased cell viability (Fig. 4b). Furthermore, HK1 overexpression significantly reversed the glucose consumption and lactate production inhibited by NAT10 knockdown (Fig. 4c‒d). These results suggested that anaerobic respiration was increased due to HK1 overexpression. Moreover, the glycolysis in RB cell lines was partly recovered through the elevation of ECAR and the decline of OCR due to HK1 overexpression (Fig. 4e‒f). To sum up, HK1 overexpression reversed the inhibition of glycolysis mediated by NAT10 knockdown.

### Knockdown of NAT10 retards tumor growth in a mouse model

**Fig. 5:**
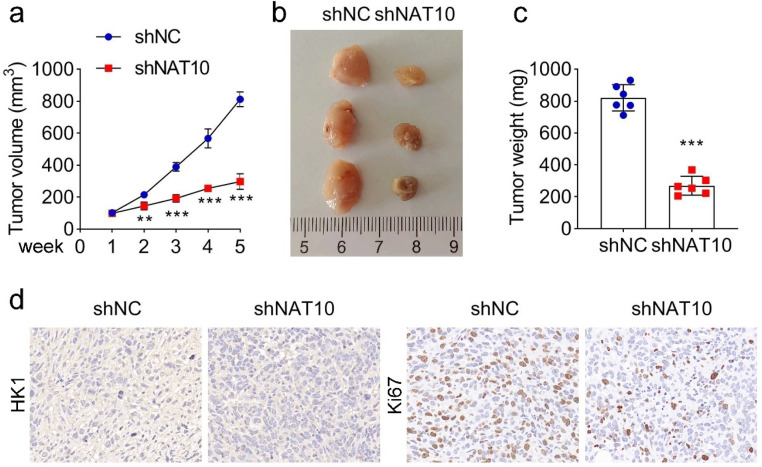
**The effect of NAT10 knockdown on RB tumor growth *in vivo*.** (a) Tumor volume from 1 to 5 weeks in mice after injecting Y-79 cells (The first week was considered when the tumor volume reached 100 mm^3^). (b) Tumor size and (c) tumor weight with or without NAT10 knockdown. (d) The expression of HK1 and Ki67 was identified by immunohistochemistry staining. ** *p* < 0.01 and *** *p* < 0.001 vs. shNC.

Finally, the authors constructed a mouse model to test the effect of NAT10 knockdown on the progression of RB *in vivo*. RB cell line Y-79 was injected into mice. The results showed that NAT10 knockdown retarded the growth of RB tumors. The tumor volume (Fig. 5a‒b) and weight (Fig. 5c) were decreased significantly with NAT10 knockdown. Moreover, the IHC assay suggested that the knockdown of NAT10 decreased the expression of HK1 and the positive rate of Ki67 (Fig. 5d). In conclusion, NAT10 knockdown retarded tumor growth in the mouse model.

## Discussion

In this study, the authors demonstrated that the expression of ac^4^C and NAT10 was elevated in RB. Moreover, the present results illustrated that NAT10 regulated RB progression by interacting with HK1 to affect glycolysis. In addition, *in vivo* experiment proved that NAT10 knockdown inhibited the growth of tumors and decreased the expression of HK1.

RB is a type of intraocular tumor attacked in early childhood that has a high fatality rate, but it is curable if diagnosed at earlier stages.^20^ Therefore, research on the etiopathogenesis of RB may provide a new strategy for early diagnosis and subsequent treatment to improve patient survival.

According to previous studies, ac^4^C modification promotes the stability of RNA and enhances translation efficiency, which is catalyzed by the acetyltransferase NAT10.^21^ Notably, ac^4^C modification accelerates the procession of multiple cancers by promoting RNA stability. Deng et al.^22^ demonstrated that ac^4^C modification mediated by helicobacter pylori-induced NAT10 promoted the stability of MDM2 mRNA to facilitate gastric cancer progression. Zheng et al.^17^ proved that NAT10 improved the stability of ac^4^C-acetylated FSP1 mRNA and enhanced its expression in colon cancer cells. However, the mechanism of ac^4^C modification on the progression of RB has never been reported yet. In the present study, the authors revealed for the first time the presence of ac^4^C modification in RB, and demonstrated that ac^4^C and NAT10 were highly expressed in both RB tissues and RB cell lines, indicating that NAT10 may be involved in RB progression by mediating ac^4^C modification.

The growth of cancer cells is based on reprogramming their metabolism. It is particularly dependent on glycolysis, which increases glucose uptake during metabolism and ferments glucose into lactic acid to meet the anabolic needs of cancer cell proliferation.^23^ Therefore, studies on the mechanism of glycolysis in RB may provide an effective therapy for treatment. A study has shown that leucine-rich pentatricopeptide repeat-motif-containing protein regulates metastasis and glycolysis by modulating autophagy and the ROS/HIF1-α pathway in RB.^24^ However, there are few studies on the regulation of ac^4^C on glycolysis. Yang et al.^25^ proved that ac^4^C promoted glycolysis addiction in gastric cancer via NAT10/SEPT9/HIF-1α positive feedback loop, and the lack of ac^4^C inhibited tumor growth *in vivo*. In this study, the authors demonstrated that NAT10 knockdown decreased glucose consumption and lactate production and inhibited glycolysis, whereas the inhibition was reserved by overexpressed HK1, suggesting that NAT10 regulated glycolysis by modulating HK1 expression. HK1 one of the HK enzymes known to regulate glucose metabolism,^26^ has been reported to affect levels of glycolysis in multiple cancers.^27^, ^28^, ^29^ In the previous study, HK1 was known to mediate a metabolic switch from OXPHOS to glycolysis to regulate the progression of RB.^6^ Research about ac^4^C modification of HK1 has not been reported yet. The present study illustrated that NAT10 enhanced the stability of HK1 mRNA by regulating the ac^4^C modification of HK1, suggesting that NAT10 knockdown inhibited glycolysis in RB cells by remodeling ac^4^C modification of HK1, thus attenuating RB progression. The mechanism of ac^4^C modification on RB development was demonstrated for the first time.

However, limitation still exists in this study. The authors only found the ac^4^C modification on HK1. Whether ac^4^C modification on other mRNAs regulates RB progression remains unclear, which may be conducted in future experiments.

In conclusion, the authors revealed that NAT10-mediated ac^4^C modification promotes the progression of RB by improving the HK1 mRNA stability to enhance glycolysis, which suggested that targeted regulation of ac^4^C modification may be an effective therapy approach for the treatment of RB.

## Ethics approval and consent to participate

This study was performed in line with the principles of the Declaration of Helsinki. Approval was granted by the Ethics Committee of Yantai Yuhuangding Hospital (Approval no. 2025-075). Written informed consent was obtained from the legal guardians. All animal experiments should comply with the ARRIVE guidelines. All methods were carried out in accordance with relevant guidelines and regulations.

## Consent for publication

Not applicable.

## Availability of data and materials

The datasets used and/or analyzed during the current study are available from the corresponding author upon reasonable request.

## Authors’ contributions

All authors contributed to the study's conception and design. Material preparation, data collection and analysis were performed by XL and FJ. The first draft of the manuscript was written by SX and all authors commented on previous versions of the manuscript. All authors read and approved the final manuscript.

## Funding

The authors declare that no funds, grants, or other support were received during the preparation of this manuscript.

## Declaration of competing interest

The authors declare no conflicts of interest.
